# Editorial: Theory-based e-mental health interventions for improving the mental and physical health of people with diabetes

**DOI:** 10.3389/fcdhc.2025.1767976

**Published:** 2026-01-08

**Authors:** Giulia Bassi, Silvia Spaggiari, Edith Eva Holloway, Daniela Di Riso

**Affiliations:** 1 Department of Developmental and Socialization Psychology, University of Padova, Padova, Italy; 2 Australian Centre for Behavioural Research in Diabetes (ACBRD), Melbourne, VIC, Australia

**Keywords:** diabetes, e-mental health, implementation fidelity, motivation, quality of life, self-management

Diabetes is a chronic condition that affects not only physical health but also psychological well-being across the lifespan. Both adults and adolescents living with type 1 (T1D) and type 2 diabetes (T2D) experience elevated rates of affective distress, including anxiety, depression, and diabetes-related distress, which can interfere with treatment adherence, glycemic levels, self-efficacy, and overall quality of life. A growing body of evidence has shown that psychological burden is closely related to maladaptive diabetes self-management behaviors and negative metabolic outcomes, reinforcing the need for integrated approaches that address both the medical and psychosocial dimensions of diabetes care.

In recent years, e-mental health interventions have gained increasing attention as scalable and accessible tools to support people with diabetes. Digital solutions, such as mobile applications, web-based platforms, telehealth programs, and remote monitoring systems, provide new opportunities to deliver theory-based psychological, medical and behavioral support beyond traditional clinical care. Cognitive Behavioral Therapy, mindfulness-based approaches, motivational techniques, and self-regulation frameworks have all been translated into digital formats with promising results. However, despite rapid innovation, evidence remains heterogeneous, particularly regarding the psychosocial effectiveness of these interventions, the mechanisms through which they operate, and the conditions under which they are most beneficial.

This Research Topic was designed to address these gaps by focusing specifically on theory-based e-mental health interventions for adults and adolescents with diabetes. By bringing together studies grounded in established psychological theories and spanning different methodological approaches, this Topic seeks to clarify not only whether digital interventions work, but *how* and *for whom* they work.

The Research Topic presents 3 contributions. First, a systematic review and meta-analysis evaluating the impact of eHealth interventions on adolescents with diabetes, with a particular emphasis on psychosocial outcomes. This work demonstrated that eHealth interventions yielded moderate and significant improvements in overall quality of life and satisfaction with life - a subscale of quality of life - among adolescents with T1D. Effects on glycemic management were smaller and non-significant. Notably, interventions were found to be well accepted, with no significant differences in dropout rates between intervention and control groups. These findings underscore the importance of explicitly targeting affective well-being, including diabetes distress, and quality of life, by viewing these outcomes as equal to the monitoring of glycemic management.

A second article contributed an original empirical investigation into the motivational foundations of health technology use among adults with T2D. Drawing on self-determination theory and the Motivation, Engagement, and Thriving in User Experience (METUX) model, this study showed that technology use is not inherently beneficial. Instead, the quality of motivation underlying technology adoption plays a central role. While general technology use was associated with healthier diet and physical activity behaviors, only *autonomously motivated* technology use predicted more adaptive health behaviors among users. In contrast, controlled motivation showed weak or even negative associations. These findings provide a crucial theoretical insight for the design of digital health interventions: supporting autonomy, competence, and a sense of agency may be as important as the technological features themselves.

The third contribution addressed a complementary but equally critical dimension: implementation fidelity and cultural adaptation in digital diabetes care. Through a detailed methodological description of a diabetes tele-management program for Hispanic/Latino adults with T2D, this article illustrated how theory-based interventions can be translated into real-world clinical practice. The study emphasized key elements such as culturally congruent communication, therapeutic alliance, shared decision-making, and structured training in technology use. By foregrounding fidelity and contextual sensitivity, this study highlighted that the effectiveness of e-mental health interventions can depend not only on theoretical soundness but also on how interventions are designed, delivered, and sustained in routine care settings.

Taken together, these contributions converge on a central message: at this early stage of the evidence base, the potential value of e-mental health interventions for diabetes appears to lie in the alignment between grounded psychological theory, individuals’ motivation to engage with digital health technologies in ways that are experienced as self-endorsed, meaningful, and supportive of their own health goals, and the quality with which these interventions are implemented in real-world care settings. At the same time, their use should not lead to an exclusive reliance on digital tools or to the replacement of interpersonal and professional sources of care. Rather, e-mental health solutions are most effective when they encourage timely help-seeking and are embedded within broader systems of traditional diabetes care, functioning as complementary resources that enhance, rather than substitute, ongoing clinical support.

